# The role of miRNA in motor neuron disease

**DOI:** 10.3389/fncel.2014.00015

**Published:** 2014-01-30

**Authors:** Min Jeong Kye, Inês do Carmo G. Gonçalves

**Affiliations:** ^1^Institute of Human Genetics, University of CologneCologne, Germany; ^2^Institute for Genetics, University of CologneCologne, Germany

**Keywords:** microRNA, motor neurons, neuromuscular junction, spinal muscular atrophy, amyotrophic lateral sclerosis

## Abstract

microRNA is a subset of endogenous non-coding RNA. It binds to partially complementary sequences in mRNAs and inhibits mRNA translation by either blocking translational machinery or degrading mRNAs. It is involved in various cellular processes including cell cycle, development, metabolism, and synaptic plasticity. Dysregulation of miRNA expression and function is reported in various diseases including cancer, metabolic disorders as well as neurological disorders. In nervous system, miRNA related pathways play a very important role in development and function of neuronal cells. Moreover, numerous evidences suggest that dysregulated miRNA related pathways contribute to pathology of neurological disorders such as Alzheimer’s disease, amyotrophic lateral sclerosis (ALS) and spinal muscular atrophy (SMA). Here, we review current knowledge about the role of miRNAs in motor neuron disorders, especially about two common diseases: SMA and ALS.

## INTRODUCTION

microRNAs (miRNAs) are endogenous non-coding single-stranded RNA molecules that play important roles in eukaryotic gene expression through posttranscriptional regulation (Bartel, 2009). It mainly binds to the 3′-untranslated region (3′-UTR) of messenger RNAs (mRNAs) from target protein-coding genes and leads to gene silencing by mRNA cleavage, translational repression and deadenylation (Huntzinger and Izaurralde, 2011). Functional studies indicate that miRNA plays a significant role in a broad range of cellular and developmental processes such as stem cell maintenance (Houbaviy et al., 2003), differentiation (Chen et al., 2004; Naguibneva et al., 2006), cell cycle (Hatfield et al., 2005), development (Hornstein et al., 2005), learning and memory formation (Gao et al., 2010), energy metabolism (Gao et al., 2009), and immune responses (Xiao et al., 2007). miRNAs can only function as part of ribonucleoprotein (RNP) complex called RNA induced silencing complex (RISC), which contains various proteins such as argonautes, fragile X mental retardation protein (FMRP), Dicer and monkey leukemia virus 10 (MOV10; Jin et al., 2004; Landthaler et al., 2008). Like miRNAs, RISC plays a crucial role in many cellular processes during development and cellular differentiation. For example, deletion of core protein of RISC, Ago2 leads to embryonic lethality in mouse (Morita et al., 2007). Similarly, ablation of *Dicer* or DiGeorge syndrome critical region gene 8 (*DGCR8*) also causes developmental arrest in murine embryonic stem cells (Bernstein et al., 2003; Wang et al., 2007). Although it is widely accepted that miRNAs are important for nervous system development and function, a little is known about the role of individual miRNAs in neuron at the moment.

## BIOGENESIS AND DECAY OF miRNAs

The majority of characterized miRNAs are transcribed by RNA polymerase II from their own independent genes or introns of protein-coding genes (Krol et al., 2010). The primary transcript (pri-miRNAs) of miRNA is specifically recognized by microprocessor complex. The microprocessor complex is composed by nuclear ribonuclease III, Drosha, and its binding partner, DGCR8. In this complex, a double stranded RNA-binding protein, DGCR8 recognizes the stem-loop structure in pri-miRNA and Drosha cleaves the pri-miRNA into a ~70-nucleotide precursor form (pre-miRNA). Afterward, pre-miRNA is translocated to the cytoplasm by exportin 5 through the nuclear pore complex in a Ran guanosine triphosphate (RanGTP)-dependent process. In the cytoplasm, second RNaseIII containing protein complex cleaves pre-miRNA to ~20 bp miRNA/miRNA* duplex. This complex is composed of Dicer (RNaseIII), transactivation response RNA binding protein (TRBP), and protein activator of the interferon-induced protein kinase (PACT). One strand of the miRNA duplex is incorporated into the RISC as a mature miRNA (guide strand/miRNA), whereas the other strand (passenger strand/miRNA^*^) is degraded (Kim et al., 2009).

miRNA can only function in the RISC. The functional core proteins of RISC are mainly composed of Argonaute families (Ago1-4) and glycin-tryptophan protein of 182 kDa (GW182). In RISC, only Agonaute proteins show endonuclease enzymatic activity, which is responsible for mRNA silencing (Huntzinger and Izaurralde, 2011). GW182 proteins are also essential for the RISC function (Eulalio et al., 2008). It inhibits binding of poly-A binding protein (PABPC) to poly-A tail and induces deadenylation, decapping, and decay of mRNAs (Zekri et al., 2009). Additional proteins associated with RISC have been identified such as FMRP, MOV10, and Hu-Antigen R (HuR; Landthaler et al., 2008).

Expression of miRNAs seems to be tightly regulated by sophisticated mechanisms from the biogenesis to decay. Although it is not yet fully understood, the stability of mature miRNA is controlled by endogenous factors such as miRNA degrading enzymes and occupancy by target mRNAs. In animal cells, miRNA decay is carried out by the 5′-to-3′ exoribonuclease 1 and 2 (XRN1 and XRN2; Bail et al., 2010). Interestingly, it seems that binding to their target mRNAs can increase or decrease stability of miRNAs (Chatterjee et al., 2011; Iio et al., 2013). Additionally, environmental factors such as growth factors, cell cycle, and neuronal activity can influence on stability of miRNAs (Ruegger and Grosshans, 2012).

## miRNA IN MOTOR NEURONS

Numerous studies have shown that miRNAs play an important role for nervous system development (Conaco et al., 2006; Akerblom et al., 2012; Zhu et al., 2013). Disturbing miRNA biogenesis pathway by deleting Dicer1 from spinal motor neurons in mouse caused spinal muscular atrophy (SMA) like phenotype (Haramati et al., 2010). More specifically, modifying miR-9 expression in developing motor neurons alters motor neuron subtype specification as well as columnar development of spinal cords in chick embryos (Otaegi et al., 2011a). In this model, miR-9 regulates expression of transcription factor, forkhead box P1 (*FoxP1*), which plays a crucial role for development and neuronal subtype differentiation in spinal cord (Otaegi et al., 2011b). Another interesting miRNA in motor neuron is miR-17-3p. This miRNA directly regulates mRNA translation of oligodendrocyte transcription factor 2 (*Olig2*), which is an important transcription factor for spinal motor neuron differentiation. The expression of miR-17-3p is repressed by sonic hedgehog (Shh), which results in elevated expression of *Olig2*. Thus, high amount of Shh will direct neuronal progenitors to differentiate toward motor neurons and decrease interneuron population. Additionally, deletion of miR-17~92 cluster from mouse embryos suggests that this cluster regulates differentiation of interneurons as well as motor neurons in early spinal cord development (Chen et al., 2011). **Table 1** summarizes motor neuron related miRNAs discussed in this review.

**Table 1:** The list of motor neuron related miRNAs discussed in this review.

miRNA	Findings	Target genes	Related disease/model organism	Reference
miR-8	Presynaptic bouton formation/synaptic growth	Lar and Wg	Fruit fly	Nesler et al. (2013)
miR-9	Motor neuron subtype specification/spinal cord development	FoxP1	Chick	Otaegi et al. (2011a,b)
	Dysregulated expression in motor neurons differentiated from embryonic stem cells		SMA/mouse	Haramati et al. (2010)
	Expression is elevated in induced stem cell derived neurons from ALS patient		ALS/cell line (Human)	Zhang et al. (2013)
	Axon growth	MAP1B	Mouse	Dajas-Bailador et al. (2012)
miR-17-3p	Spinal motor neuron differentiation	Olig2	Mouse	Chen et al. (2011)
miR17~92 cluster	Differentiation of interneuron and motor neurons		Mouse	Chen et al. (2011)
miR-21	Axonal regeneration	Sprouty2	Mouse	Strickland et al. (2011)
	Spinal cord injury	PTEN and FasL	Rat	Hu et al. (2013)
miR-23a, miR-29b, and miR-455	Expression is increased in skeletal muscle from ALS patients and it may cause dysregulation in mitochondrial gene expression	PGC1-α (miR-23a)	ALS/mouse	Russell et al. (2012)
miR-106	Regeneration of NMJ	HDAC4	ALS/mouse	Williams et al. (2009)
miR-124	Neurotransmitter release		Fruit fly	Sun et al. (2012)
	Neurotransmitter release at the NMJ	Rab3a	Slow-channel congenital myasthenic syndrome/mouse	Zhu et al. (2013)
miR-125 and let-7	NMJ phenotype, delayed maturation, smaller size, abnormal locomotion, reduced lifespan	Abrupt	Fruit fly	Caygill and Johnston (2008)
miR-132	Neuronal morphology and cognition	AChE and p250GAP	ALS/mouse	Edbauer et al. (2010), Shaltiel et al. (2013)
	Splicing of Tau	PTBP2	Tauopathies/mouse	Smith et al. (2011), Hebert et al. (2012)
miR-132, miR-143, and miR-558	Expression is dysregulated in TDP-43 deficient cells. Neurites outgrowth (miR-132)		ALS and FTLD (frontotemporal lobar degeneration)/cell line (mouse)	Kawahara and Mieda-Sato (2012)
miR-133b	Spinal cord regeneration	RhoA	Zebrafish	Yu et al. (2011)
miR-134	Neuronal development and dendritogenesis	Pumilio2	Mouse	Fiore et al. (2009)
miR-138	Axonal regeneration	SIRT1	Mouse	Liu et al. (2013)
miR-310	Neurotransmitter release at the NMJ	Khc-73	Fruit fly	Tsurudome et al. (2010)
miR-338-3p	Expression is elevated in ALS patients		ALS/human	De Felice et al. (2012)
miR-431	Axonal regeneration and outgrowth	Kremen1	Mouse	Wu and Murashov (2013)
miR-451	Dysregulated in immune cells (with many other miRNAs)		ALS/mouse	Butovsky et al. (2012)

miRNAs are also important for axonal regeneration process in spinal motor neurons. Ablation of Dicer in sciatic nerve of mouse suggested that functional miRNAs are pivotal for nerve regeneration and axonal re-growth (Wu et al., 2012). In zebrafish, elevated expression of miR-133b after spinal cord injury represses mRNA translation of RhoA. This process promotes functional recovery of motor neuron axons after traumatic injury (Yu et al., 2011). Taken together, we can conclude that miRNAs play a significant role in various processes in motor neurons such as neuronal subtype specification, functional maintenance, and regeneration after injury.

## miRNA AT THE NEUROMUSCULAR JUNCTION

miRNAs are differentially distributed in neuronal compartments such as soma and neurites (Kye et al., 2007). Numerous miRNAs are presented in axonal compartment and growth cones in cortical and sympathetic neurons (Natera-Naranjo et al., 2010; Sasaki et al., 2013). These data suggests that miRNAs can regulate mRNA translation at the axonal compartments. It seems that miRNAs play a significant role in motor neuron axons, especially at the neuromuscular junction (NMJ). Interestingly, in contrast to vertebrates, *Drosophila* mutant, who lacks one of the most abundant neuronal miRNA, miR-124 did not show any strong defect in neuronal production, differentiation, and NMJ morphology. However, the miR-124 *Drosophila* mutant showed shorter life span, impaired locomotion as well as increased presynaptic neurotransmitter release at the NMJ (Sun et al., 2012). Additionally, miR-8 is also reported as an important miRNA for pre-synaptic bouton formation at the *Drosophila* NMJ. Repeated neuronal activity represses the expression of miR-8, and it results in elevated neuronal mRNA translation and synaptic growth (Nesler et al., 2013). *Drosophila* mutants lacking miR-125 or let-7 expression also showed defects in NMJ phenotypes such as delayed maturation of NMJ, smaller size of NMJ, and abnormality in locomotion. Interestingly, these phenotypes are shown only during metamorphosis (Caygill and Johnston, 2008). This implies that miRNAs play a specific role for temporal and spatial regulation of gene expression during development. Another interesting miRNA is miR-310. It regulates synaptic homeostasis at the NMJ by regulating translation of kinesin super family member, Khc-73. miR-310 directly represses the translation of Khc-73 to control neurotransmitter release in motor neurons during larval stages (Tsurudome et al., 2010). Finally, function of miRNA at mammalian NMJ is described in a mouse model for neuromuscular disease, slow-channel congenital myasthenic syndrome (SCS). Axonal expression of miR-124 is elevated in this model compared to the wild type animals. miR-124 regulates mRNA translation of *Rab3a* in axon in response to amplified Ca^2^^+^/calpain/cdk5/nitric oxide pathway in muscle cells. In consequence, the elevated expression of miR-124 and reduced expression of Rab3a proteins in nerve terminals decrease neurotransmitter release to the NMJ (Zhu et al., 2013). Taken together, we can conclude that miRNAs are important players for NMJ formation and function as well as maintaining synaptic homeostasis.

Recently, protein and nucleic acids containing vesicles (exosomes) have been suggested as a new molecular mechanism for communication between cells in nervous system (Sharma et al., 2013). They can transfer genetic molecules from donor cells to recipient cells, by which they can change physiology of recipient cells (Valadi et al., 2007). For example, cancer cells release more exosomes than healthy ones. In consequence, it changes physiology of surrounding cells to have more favorable conditions for their metastasis (Grange et al., 2011; Soldevilla et al., 2013). Moreover, various miRNAs are detected in exosomes released from neurons and muscle cells (Forterre et al., 2013; Fruhbeis et al., 2013). These findings strongly suggest that miRNA can function as a signaling molecule for intracellular communication at the NMJ.

## TWO COMMON MOTOR NEURON DISEASES; SMA AND ALS

### PROXIMAL SPINAL MUSCULAR ATROPHY

Spinal muscular atrophy is a genetically and clinically heterogeneous group of neuromuscular disorders characterized by progressive degeneration of lower alpha motor neurons in the anterior horn of spinal cord (Crawford and Pardo, 1996). Affected individuals exhibit proximal manifestation of muscle weakness and atrophy. With an incidence of 1:6000 ~ 1:10000 newborns and a carrier frequency of 1:35, proximal SMA is the leading hereditary cause of infant mortality (Wirth et al., 2006). Due to the highly variable disease severity, four clinical types of SMA are classified based on the age of onset and achieved motor abilities: Type I SMA (Werdnig–Hoffmann), intermediate Type II SMA, mild Type III SMA (Kugelberg–Welander), and Type IV SMA (adult SMA; Pearn, 1980; Wirth et al., 2013).

Survival of motor neuron 1 (*SMN1*) is the major disease-determining gene of SMA. SMA is caused by homozygous deletion or mutation of *SMN1* (Lefebvre et al., 1995). This gene is located on the chromosomal region 5q11.2-13.3 in a segment of ~500 kb, which includes the telomeric *SMN1* and the similar but slightly different centromeric *SMN2*. *SMN2,* which is >99% identical to *SMN1,* has only a reduced capacity for correct splicing due to a single silent mutation in exon7. *SMN2* produces about 10% of full-length *SMN2* RNA that encodes a protein identical to the one from *SMN1. SMN2*, which can vary from one to six copies per genome, is the main modifier of SMA and influences on SMA severity. The severity of SMA can be affected by various genetic factors (Wirth et al., 2013). The human SMN protein is a 38 kDa protein, which forms multi-protein complex with its binding partners, seven Gemins (Gemin 2–8). Self-oligomerization of SMN creates the backbone of the complex (Battle et al., 2006). SMN is ubiquitously expressed and can be found in the nucleus as well as in the axons and dendrites of neurons (Cougot et al., 2008; Akten et al., 2011). In the nucleus, SMN is localized in subcellular structures called gems, where it interacts with a number of proteins that are essential for RNA processing and splicing (Pellizzoni et al., 1998). In the axons, SMN interacts with RNA-binding proteins, such as HuD, and plays a role in RNP trafficking for local mRNA translation (Akten et al., 2011; Fallini et al., 2011). SMN also binds to FMRP (Piazzon et al., 2008), KH-type splicing regulatory protein (KSRP; Tadesse et al., 2008) and fused in sarcoma (FUS; Yamazaki et al., 2012), which are important for miRNA biogenesis and function.

### AMYOTROPHIC LATERAL SCLEROSIS

Amyotrophic lateral sclerosis (ALS) is a progressive neurodegenerative disorder, which leads to death within 2–3 years of onset. It is one of the most common motor neuron diseases occurring 1.7 ~ 2.3 out of 100,000 person per year in worldwide (Beghi et al., 2006). The symptom of ALS usually starts after age 50, but it can happen in younger age group. The disease manifests itself by onset of degeneration in specific subset of motor neurons. It progressively spreads to neighboring motor neurons and leads to atrophy of associated muscle tissues (Pratt et al., 2012). The genetic and environmental causes of ALS is still under investigation, but 90% of them are sporadic or from unknown genetic factors. So far only about 10% of the cases can be traced to genetic factors (Al-Chalabi et al., 2012). The most well known genetic cause of ALS is mutations in or deletion of Cu/Zn Super Oxide Dismutase 1 (*SOD1*; Rosen et al., 1993). Only recently, with advanced genomic screening tools, several other genes associated with ALS have been identified including TAR DNA-binding protein (*TDP-43*), *FUS*, ALS 2 (*ALS2*), neurofilament heavy peptide (*NEFH*; Al-Chalabi et al., 2012) and C9ORF72 (DeJesus-Hernandez et al., 2011; Renton et al., 2011). However, the cellular and pathological mechanisms causing ALS due to mutation or deletion of these genes are still under investigation.

## miRNA IN MOTOR NEURON DISEASES

The role of miRNA and their target genes are intensively studied in cancer field. Studies in cancer cells suggest us that miRNA are involved in many different pathways and their dys-regulation can cause various types of cancers (Esteller, 2011; Jansson and Lund, 2012). While the role of individual miRNAs in neurological disorders is not yet fully understood, there are growing evidences that miRNAs play a critical role in neurological disorders, such as miR-206/miR-153 in Alzheimer’s disease (Lee et al., 2012; Liang et al., 2012), miR-34b/miR-9/miR-9^*^ in Huntington’s disease (Packer et al., 2008; Gaughwin et al., 2011), miR-128a/miR-24/let-7b in mood disorder (Zhou et al., 2009), miR-189 in Tourette’s syndrome (Abelson et al., 2005), miR-9 in SMA (Haramati et al., 2010), miR-106/miR-338-3p/miR-451 in ALS (Williams et al., 2009; Butovsky et al., 2012; De Felice et al., 2012), miR-21/miR-431/miR-138 for axonal regeneration for sensory neurons (Strickland et al., 2011; Liu et al., 2013; Wu and Murashov, 2013), and miR-133b/miR-21 for spinal cord injury (Yu et al., 2011; Hu et al., 2013). However, pathological contribution of individual miRNAs to each of these diseases is still under investigation. Especially, our knowledge about the role of miRNAs in motor neuron diseases is very limited. This can be both due to the complexity of the nervous system and the technical difficulties of studying neurological disorders. In this section, we will focus on miRNA related dysregulation in two motor neuron diseases; SMA, and ALS.

### miRNA IN SPINAL MUSCULAR ATROPHY PATHOLOGY

As it is mentioned above, SMN is a RNA binding protein, which forms a complex with other Gemin proteins, Gemin2-8 (Battle et al., 2006). Among the Gemins, Gemin3 and Gemin4 also bind to Ago2, which served the core protein in RISC and plays a role in miRNA biogenesis (Mourelatos et al., 2002). Moreover, numerous miRNAs bind to Gemin3 in human and murine neuronal cell lines (Dostie et al., 2003). From these reports, we can reason that the SMN complex is involved in miRNA biogenesis and/or function. In fact, expression of miRNAs such as miR-9 and miR-9* were dysregulated in murine embryonic stem cell derived motor neurons harboring a mutation causing SMA (Haramati et al., 2010). However, cellular mechanisms underlying in SMN mediated miRNA expression and/or function is not yet identified.

Dicer is an RNase playing a role in miRNA biogenesis pathway. The enzyme acts on the stem-loop shape of precursor miRNA (pre-miR) and creates a doubles stranded-miRNAs by cleaving the loop structure off from the pre-miR (Bernstein et al., 2001). It seems that a group of miRNAs get mature by Dicer, while other subset of miRNAs are processed by Ago2 (Cheloufi et al., 2010). Thus, deletion of either Dicer or Ago2 protein in cell may lead to severe impairments in miRNA biogenesis and function. In fact, deletion of Dicer in post-mitotic motor neuron causes motor neuron degeneration, similar to the neuromuscular phenotype observed in mouse model for SMA (Haramati et al., 2010).

### miRNA IN AMYOTROPHIC LATERAL SCLEROSIS PATHOLOGY

Dysregulation in miRNA expression and miRNA-related pathways are also reported in ALS. The expression of miR-206, a skeletal muscle specific miRNA, was increased in muscle after denervation of sciatic nerve and its deficiency synergistically worsened disease progress in ALS mouse model harboring a disease causing mutation in superoxide dismutase, *SOD1*. In this study, the elevated expression of miR-206 after denervation promotes reinnervation process at the NMJ via regulating histone deacetylase 4 (*HDAC4*) and fibroblast growth factor (FGF) pathway (Williams et al., 2009). This study is the first report suggesting the role of miRNA for nerve regeneration at the NMJ. Recently, additional report showed that the expression of miR-23a, miR-29b, and miR-455 are elevated in skeletal muscle tissues from ALS patients and this may cause dysregulation in mitochondrial gene expression (Russell et al., 2012). These findings hint us that dysregulated expression of miRNA in muscle cells significantly contributes to ALS pathology.

Another ALS associated gene, TAR DNA-binding protein-43 (TDP-43) is directly involved in miRNA pathway. TDP-43 is a component of the Dicer and Drosha complexes, which are important for miRNA biogenesis. TDP-43 selectively regulates pri-miRNA processing by binding to primary transcripts of specific miRNAs. It is reported that mutations in *TDP-43* gene causes differential expression of mature and functional miRNAs such as miR-132, miR-143 and miR-558 that in turn contribute to ALS pathology. Interestingly, TDP-43 deficiency caused impairment in neurite outgrowth in Neuro2a cells and it was rescued by over-expressing miR-132 (Kawahara and Mieda-Sato, 2012). Additionally, elevated expression of miR-9 is also observed in induced pluripotent stem cell-derived neurons from ALS patient harboring a mutation in *TDP-43* (Zhang et al., 2013). Together, these data suggest that TDP-43 is required for neuronal differentiation and neurite outgrowth via regulating miRNA biogenesis pathway and their expression.

Mutations in RNA binding protein FUS/TLS (FUS/translocated in liposarcoma) are also found in ALS patients (Kwiatkowski et al., 2009). Similar to TDP-43, FUS/TLS protein binds to pre-mRNA molecules and determines their fate via regulating splicing, transport, stability, and translation (Lagier-Tourenne et al., 2012). Recently, it has been shown that FUS/TLS promotes biogenesis of specific miRNAs via recruiting Drosha to primary miRNA transcripts. Among them, miRNAs with known function for synaptic plasticity and neuronal development such as miR-132, miR-134, and miR-9 were identified (Morlando et al., 2012). miR-9 regulates axon growth via direct regulation of microtubule-associated protein 1b (MAP1B) mRNA translation and miR-132 regulates neuronal morphology and growth by targeting various genes including acetylcholinesterase and Tau (Edbauer et al., 2010; Smith et al., 2011; Dajas-Bailador et al., 2012; Hebert et al., 2012; Shaltiel et al., 2013). miR-134 regulates neuronal development and dendritogenesis in response to neuronal activity (Fiore et al., 2009). These results suggest that mutations in FUS/TLS may lead to disturbance in miRNA biogenesis and function, which contributes to pathological phenotype observed in ALS patients.

In addition to it, miRNA expression in white blood cells from sporadic ALS patients exhibited distinct expression pattern during disease progress. miRNA profiling data from 14 patients and 14 controls showed that expression of miR-338-3p is increased and expression of seven other miRNAs is decreased in leukocyte from ALS patients (De Felice et al., 2012). Since bloods are more accessible than motor neurons from patients, this finding suggests that profiling of miRNA expression from bloods can be used as a diagnostic tool for ALS. Taken together, we can conclude that dysregulated miRNA expression contributes to the ALS pathology and profiling of the miRNA expression can serve as a tool for ALS diagnosis.

## SUMMARY AND PERSPECTIVE

With current advanced genetic tools, genetic causes of motor neuron diseases are getting unveiled. However, pathogenesis of motor neuron disease is extremely complex and not yet fully understood. Due to this reason, efficient cure for motor neuron disease is currently unavailable. Interestingly, even though the genetic causes of the diseases are different, their cellular pathomechanisms share common traits; miRNA biogenesis and expression. Here, we reviewed current knowledge about the role of miRNA in two common motor neuron diseases, SMA and ALS. Dysregulated miRNA expression in neuromuscular system may lead to neurodegeneration and disease pathology. However, the question, how individual miRNAs contribute to development and maintenance of the NMJ and how their dysregulation may cause ALS or SMA, requires further investigation.

## Conflict of Interest Statement

The authors declare that the research was conducted in the absence of any commercial or financial relationships that could be construed as a potential conflict of interest.
